# Recruitment of dendritic cells and macrophages during T cell-mediated synovial inflammation

**DOI:** 10.1186/ar2328

**Published:** 2007-11-20

**Authors:** Mahin Moghaddami, Leslie G Cleland, Gorjana Radisic, Graham Mayrhofer

**Affiliations:** 1 Arthritis Research Laboratory, Hanson Research Institute, Institute of Medical and Veterinary Science, Frome Road, Adelaide, South Australia, 5000, Australia; 2 Rheumatology Unit, Royal Adelaide Hospital, North Terrace, Adelaide, South Australia, 5000, Australia; 3 Discipline of Microbiology and Immunology, School of Molecular and Biomedical Science, University of Adelaide, North Terrace, Adelaide, South Australia, 5005, Australia

## Abstract

Adoptive transfer of adjuvant-induced arthritis was used in this study to examine local macrophages and dendritic cells (DCs) during T cell-mediated synovial inflammation. We studied the influx of CD11b^+^CD11c^+ ^putative myeloid DCs and other non-lymphoid CD45^+ ^cells into synovium-rich tissues (SRTs) of the affected hind paws in response to a pulse of autoreactive thoracic duct cells. Cells were prepared from the SRTs using a collagenase perfusion-digestion technique, thus allowing enumeration and phenotypic analysis by flow cytometry. Numbers of CD45^+ ^cells increased during the first 6 days, with increases in CD45^+^MHC (major histocompatibility complex) II^+ ^monocyte-like cells from as early as day 3 after transfer. In contrast, typical MHC II^- ^monocytes, mainly of the CD4^- ^subset, did not increase until 12 to 14 days after cell transfer, coinciding with the main influx of polymorphonuclear cells. By day 14, CD45^+^MHC II^hi ^cells constituted approximately half of all CD45^+ ^cells in SRT. Most of the MHC II^hi ^cells expressed CD11c and CD11b and represented putative myeloid DCs, whereas only approximately 20% were CD163^+ ^macrophages. Less than 5% of the MHC II^hi ^cells in inflamed SRT were CD11b^-^, setting a maximum for any influx of plasmacytoid DCs. Of the putative myeloid DCs, a third expressed CD4 and both the CD4^+ ^and the CD4^- ^subsets expressed the co-stimulatory molecule CD172a. Early accumulation of MHC II^hi^CD11c^+ ^monocyte-like cells during the early phase of T cell-mediated inflammation, relative to typical MHC II^- ^blood monocytes, suggests that recruited monocytes differentiate rapidly toward the DC lineage at this stage in the disease process. However, it is possible also that the MHC II^hi^CD11c^+ ^cells originate from a specific subset of DC-like circulating mononuclear cells.

## Introduction

Dendritic cells (DCs) differentiate from different progenitors, with lymphoid or plasmacytoid DCs (pDCs) arising from a common lymphoid progenitor and myeloid DCs (mDCs) sharing a common lineage with monocytes and macrophages (Mϕ) [1,2]. Myeloid DCs can arise from lineage-committed circulating precursors [3,4], from monocytes [5], or from a specific subset of monocytes [6]. The function of mDCs in initiating immune responses and their potential role in maintenance of peripheral tolerance of T cells have been comparatively well studied [7,8]. However, less is known about interactions between effector T cells and DCs at sites of T cell-mediated inflammation. The DC life cycle often is described in terms of acute infection, where immature cells differentiate in response to pathogen-associated recognition patterns and migrate to the regional lymph nodes carrying microbial antigens [7,9]. However, at sites of chronic T cell-mediated inflammation, maturation of DCs appears to involve antigen-specific effector T cells and the cells remain locally [10,11], thus focusing the inflammatory response.

In an autoimmune disease such as rheumatoid arthritis, the presence of large numbers of activated DCs in the affected synovium [12,13] suggests that these cells present local autoantigens to cognate effector T cells *in situ *[14,15]. However, studies on pathological specimens present a static picture, usually of established disease, and there is little information available about the kinetics of recruitment of DC precursors in the early rheumatoid lesion. Adjuvant-induced arthritis (AA) provides a robust system in which to study the effector phase of T cell-mediated inflammation [16-18]. Nine days after inoculation of Dark Agouti (DA) strain rats with complete Freund's adjuvant (CFA), the thoracic duct (TD) lymph contains CD4^+ ^T effector cells that transfer AA to syngeneic recipients adoptively, without co-transfer of antigen-presenting cells (APCs) [18]. The donor T cells accumulate selectively in synovial tissues and their arthritogenicity and local proliferation suggest that they respond to cognate antigen(s) presented by APCs in the affected synovium [19].

Recently, we studied potential APCs in synovium-rich tissues (SRTs) prepared from healthy rats using a collagenase perfusion technique [20,21]. We identified a subset of endocytic 'indeterminate cells' that resemble mDCs. These cells expressed high levels of surface major histocompatibility complex (MHC) class II molecules but neither CD11c (DC lineage marker) nor CD163 (Mϕ marker). The fate of these cells is unknown, but *in vitro *they have the potential to differentiate into typical DCs in the presence of granulocyte-macrophage colony-stimulating factor [20]. In the present study, we used adoptive transfer of AA to investigate the effects of a pulse of pathogenic T cells on recruitment of mDC-like cells to inflamed synovial tissues. Data are presented also on recruitment of other blood-derived non-lymphoid cells, including monocytes, Mϕ, polymorphonuclear (PMN) leukocytes, and a population of CD11b^- ^mononuclear cells. T cell-induced inflammation is accompanied by increases in all of these cell types but especially in cells with the phenotypic characteristics of early DCs.

## Materials and methods

### Animals

Female inbred specific pathogen-free DA strain rats (6 weeks old) were obtained from the Gilles Plains Animal Resource Centre (Adelaide, Australia). Experimental protocols were approved by the ethics committees of the Institute of Medical and Veterinary Science and the University of Adelaide.

### Immunological reagents

Monoclonal antibodies (mAbs) are listed in Table 1[20,22,23]. Conjugated isotype-matched control mAbs, fluorescein isothiocyanate (FITC)-conjugated goat anti-mouse immunoglobulin (Ig), biotin-conjugated goat anti-mouse Ig, FITC-streptavidin, and phycoerythrin (PE)-Cy-7-streptavidin were obtained from BD Pharmingen (San Diego, CA, USA).

**Table 1:** Monoclonal antibodies

Molecule	Monoclonal antibody	Reference/Source
CD11a	WT1 – Hybridoma supernatant	[20]
CD11b	WT5 – FITC	BD Pharmingen (San Diego, CA, USA)
CD11c	8A2 – Purified	AbD Serotec (Oxford, UK)
CD4	OX35 – Cy-chrome	BD Pharmingen
CD5	OX19 – Hybridoma supernatant	[22]
CD32	D34-485 – Purified	BD Pharmingen
CD36	UA009	[23]
CD45RC	OX22 – Hybridoma supernatant	[22]
CD45R (B220)	His 24 – Purified	BD Pharmingen
CD54	IA29 – Hybridoma supernatant	[20]
CD80	3H5 – Hybridoma supernatant	[20]
CD86	24F – Purified	[20]
CD90	OX7 – Hybridoma supernatant	[22]
CD163	ED2 – Purified	AbD Serotec
CD172a	OX41 – Hybridoma supernatant	[20]
MHC II	OX6 – Phycoerythrin	BD Pharmingen
IgG1	IB5 – Hybridoma supernatant	[20]
IgG2a	ID4.5 – Hybridoma supernatant	[20]

CD, cluster of differentiation; FITC, fluorescein isothiocyanate; Ig, immunoglobulin; MHC, major histocompatibility complex.

### Induction of adjuvant-induced arthritis

Rats (7 weeks old) received 0.1 mL of CFA subcutaneously at the base of the tail [18]. Essentially all DA rats developed polyarthritis after this treatment.

### Assessment of arthritis

Arthritis in each paw was assessed as follows: 0, no evidence of arthritis; 1, a single focus of inflammation; 2, more than one focus of inflammation; 3, confluent but not global swelling; and 4, severe global swelling of the entire paw. Therefore, the disease score for individual rats ranges between 0 and 16.

### Adoptive transfer of arthritis

Arthritis was transferred adoptively to naive 7-week-old syngeneic rats by intravenous injection of 2 × 10^8 ^TD cells obtained from donors 9 days after inoculation of CFA [18]. Such donors are referred to throughout as 'arthritic donors'. To assess severity of arthritis and to prepare cells from hind-paw SRT, groups of five rats were examined at days 3, 6, 9, 12, and 14 after adoptive transfer. Five healthy rats were included as controls.

### Isolation of cells from synovium-rich tissue

Single-cell suspensions were obtained from the SRT of the hind paws by a collagenase perfusion technique, as described previously [20]. The viability of the leukocytes obtained from SRT was routinely greater than 95% by exclusion of Trypan blue.

### Immunofluorescence staining of cells

Staining for dual-color immunofluorescence was performed on aliquots of 1 to 2 × 10^5 ^cells by means of a combination of indirect and direct techniques [20].

For four-color analysis, cells were first labelled indirectly using purified mAb, followed by biotin-conjugated goat anti-mouse Ig secondary antibody and streptavidin PE-Cy-7. After the blocking of free valences by incubation with 20 μL of normal mouse serum (NMS) for 20 minutes, the cells were incubated with a cocktail of conjugated mAbs containing PE-OX6, FITC-WT5, and Cy-chrome-OX35 and were washed and fixed with 1% paraformaldehyde.

### Flow cytometry

Labelled cells were analysed with a Beckman Coulter EPICS XL-MCL flow cytometer and Coulter EXPO 32 software (Beckman Coulter, Fullerton, CA, USA). An electronic 'DC gate' was based on the light-scatter properties of DCs from rat pseudo-afferent lymph [20]. Populations of cells expressing each cell surface marker were examined by analysis of at least 50,000 cells within this gate. Absolute cell numbers were estimated by including known numbers of FITC-conjugated BD CaliBRITE beads (BD Biosciences, San Jose, CA, USA) in each sample [19].

### Cell sorting

Cells from SRT were stained to detect either CD45 (FITC-OX1, direct) and MHC class II (PE-OX6, direct), or CD11b (FITC-WT5, direct), MHC class II (PE-OX6, direct), and CD4 (PE-CY5-OX35, direct). They were sorted using FACSDiva software (BD Biosciences, San Jose, CA, USA) [20].

### Immunocytochemistry and immunohistochemistry

Cytospin smears prepared from sorted cells were fixed and stained essentially as described [20]. The primary antibodies used for sorting were saturated with anti-mouse IgG1. The smears then were incubated with undiluted NMS to block free valences and were incubated further with anti-CD11c or anti-CD163 antibodies or with isotype-matched irrelevant antibodies. Bound anti-CD11c (IgG2a) or anti-CD163 (IgG1) then was detected with biotinylated goat anti-mouse IgG2a or IgG1, respectively, followed by streptavidin-peroxidase. Bound peroxidase was detected as described previously [20]. For immunohistochemistry, inflamed tissue containing synovium was pared from the lateral side of the ankle and embedded in OCT [19]. Frozen sections (5 μm) were fixed and stained by the indirect immunoperoxidase technique as described elsewhere [20].

### Statistical analysis

Differences between mean cell numbers in experimental groups were analysed where appropriate using one-way analysis of variance, with post-analysis by the Tukey-Kramer multiple comparisons test. A *p *value of less than 0.05 between groups was considered significant.

## Results

### Cell recovery from synovium-rich tissue during adoptively transferred arthritis

**Figure 1:**
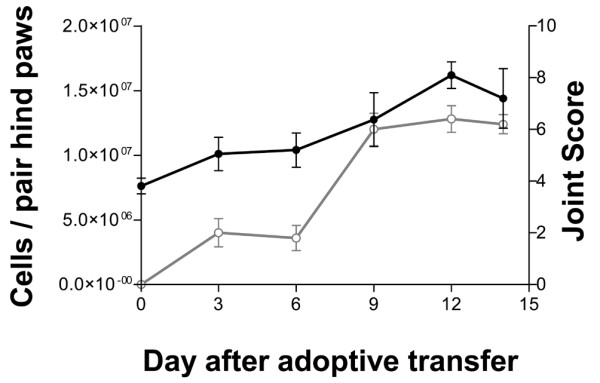
Viable cells recovered from hind paws of rats with adoptively transferred adjuvant-induced arthritis and joint scores. Rats received 2 × 10^8 ^thoracic duct cells obtained from syngeneic donors 9 days after inoculation with complete Freund's adjuvant. At the times indicated, arthritis was scored in all paws and cells then were prepared from the synovium-rich tissues of the hind paws by intra-arterial perfusion with collagenase (Materials and methods). Black indicates total viable cells per pair of hind paws (mean ± standard deviation [SD], *n *= 5). Gray indicates joint score (mean ± SD, *n *= 5).

Recipients received 2 × 10^8 ^TD cells from arthritic donors by intravenous injection. Consistent with previous findings [18], mild transient inflammation was observed at day 3 after adoptive transfer and sustained paw swelling commenced from day 6, reaching a maximum at day 12 (Figure 1). Total viable cells recovered from each pair of hind paws by collagenase digestion had increased by day 3 after adoptive transfer and reached approximately 1.5 × 10^7 ^between days 9 and 14. Thus, cell yields and arthritis severity followed similar time courses.

### Dual-fluorochrome analysis of CD45^+ ^cells prepared from synovium-rich tissue

**Figure 2:**
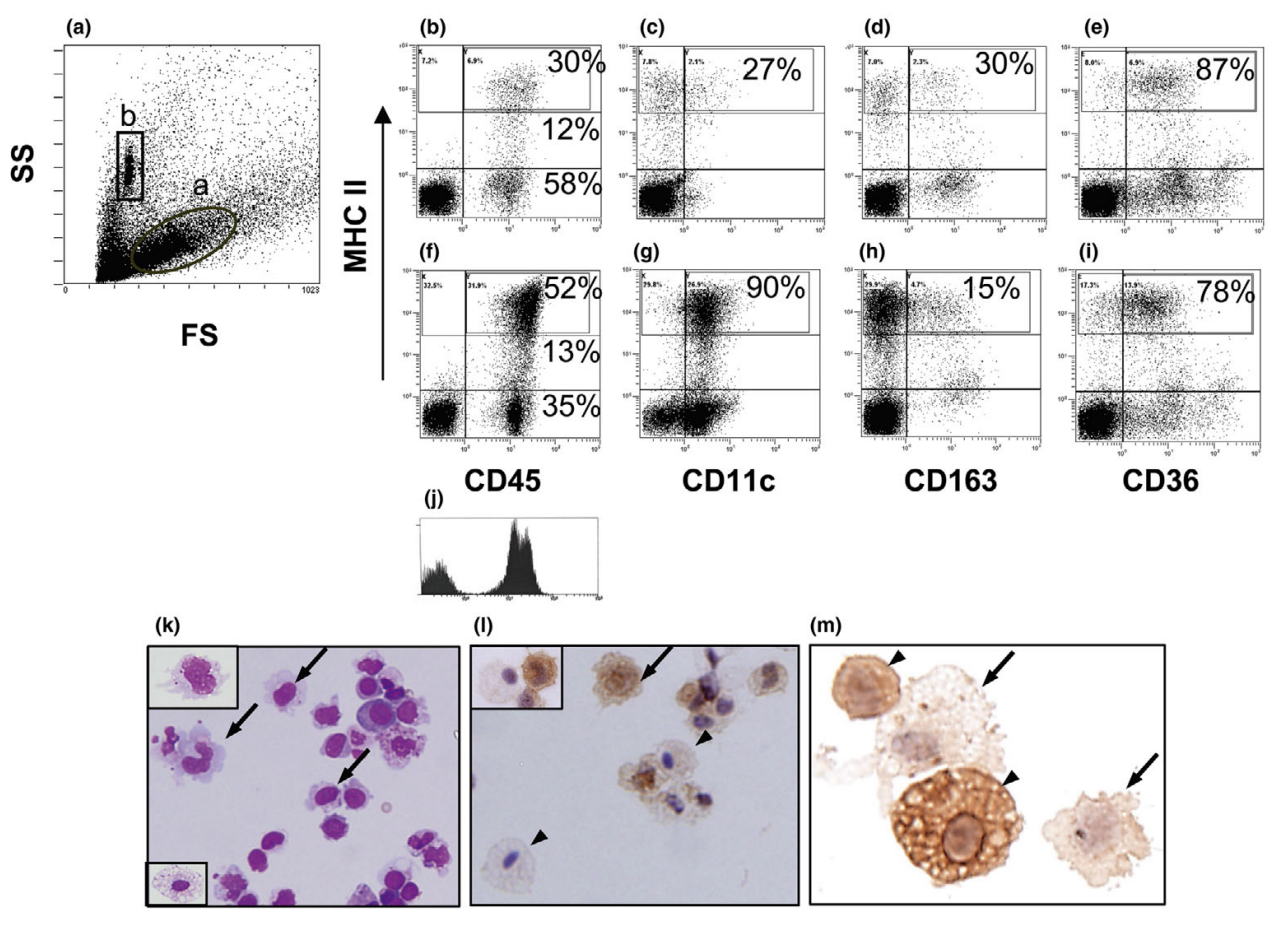
Dual-color flow cytometry and cytospin preparations of sorted CD45^+^MHC II^hi ^cells from synovium-rich tissue (SRT) of hind paws. **(a) **Forward scatter (FS) and side scatter (SS) of light, showing a gate ('dendritic cell [DC] gate') based on the light scatter of DCs in rat pseudo-afferent lymph (a) and a gate containing the CaliBRITE beads used to calculate absolute numbers of cells (b). Cells expressing MHC class II molecules in SRT from a healthy rat **(b-e) **and from a rat 14 days after adoptive transfer of arthritis **(f-i)**. All MHC II^+ ^cells express CD45 **(b, f)**. However, when CD45 events in **(f) **are plotted as a histogram **(j)**, the MHC II^- ^events (predominantly polymorphonuclear cells) express lower levels of CD45 than the MHC II^hi ^events (mononuclear cells). After adoptive transfer, more MHC II^hi ^cells express CD11c **(c, g) **and CD36 **(e, i) **but a minority express CD163 **(d, h)**. Percentages indicate proportions of CD45^+ ^cells that express indicated levels of MHC class II molecules **(b, f) **or MHC II^hi ^cells that express CD11c **(c, g)**, CD163 **(d, h)**, or CD36 **(e, i)**. Morphology of CD45^+^MHC II^hi ^cells from SRT obtained 12 days after adoptive transfer of arthritis. The cells were sorted by fluorescence-activated cell sorting, and smears were prepared by cytospin **(k-m)**. Giemsa stain shows that most cells have DC morphology (arrows), a few have veils (top inset), and a few (lower inset) have macrophage morphology **(k)**. Indirect immunoperoxidase staining shows that cells with DC morphology (arrow and inset), but not macrophage morphology (arrowheads and inset), express CD11c **(l)**. DCs did not express CD163 (arrows) but this antigen was expressed strongly by macrophages (arrowheads) **(m)**. Objective, × 60. MHC, major histocompatibility complex.

The forward and side scatter of light by cells in an SRT preparation is shown in Figure 2a. Gate 'a' (the 'DC gate') is based on the light scatter of afferent lymph DCs (Materials and methods). In addition to containing DCs, this gate contains monocytes, PMN cells, fibroblasts, and endothelial cells but excludes essentially all lymphocytes (M. Moghaddami, L.G. Cleland, G. Radisic, G. Mayrhofer, unpublished data). All analyses described below were confined to cells in the 'DC gate', whereas gate 'b' contained the fluorescent beads used for measurement of cell numbers (see below).

Cells from SRT (*n *= 5 for each time point) were analysed first by dual-fluorochrome flow cytometry (Figure 2b–i). When cells from healthy rats were labelled with mAbs against CD45 (OX1, indirect FITC) and MHC class II (OX6, PE conjugate), the CD45^+ ^cells within the DC gate were found to consist of MHC II^hi^, MHC II^lo^, and MHC II^- ^subpopulations (Figure 2b). The MHC II^hi ^and MHC II^lo/int ^subsets comprised approximately 30% and 12%, respectively, of the total CD45^+ ^cells in SRT from normal rats (Figure 2b). We have shown previously that the MHC II^hi ^cells in healthy SRT consist mainly of CD11c^-^CD163^- ^'indeterminate cells' and CD163^+^-activated Mϕ, whereas MHC II^lo/int ^cells consist mainly of CD163^+ ^Mϕ [20]. SRT preparations from healthy rats contained very few PMN cells.

A distinct subset of CD45^lo ^MHC II^- ^cells was present at 14 days after adoptive transfer (Figure 2f). This population is seen clearly when CD45^+ ^events in Figure 2f are plotted as a histogram (Figure 2j). Cytospin preparations of sorted CD45^lo^MHC II^- ^cells showed that greater than 80% were PMN cells (not shown). In contrast, the CD45^hi ^cells incorporated the MHC II^hi ^and MHC II^lo/int ^subsets and, when sorted, consisted entirely of mononuclear cells (not shown). CD45^hi^MHC II^hi ^cells comprised 52% of the CD45^+ ^cells at this time, compared with 30% in healthy rats. The sorted CD45^hi^MHC II^hi ^cells were heterogeneous in morphology (Figure 2k). Approximately 70% resembled DCs at various stages of differentiation and approximately 2% had veiled morphology (Figure 2k, upper inset). Of the remaining cells, approximately 20% resembled monocytes and 8% had the morphology of Mϕ (Figure 2k, lower inset). When stained by the indirect immunoperoxidase technique, the cells with Mϕ morphology were found to express CD163 (Figure 2m) but not CD11c (Figure 2l and inset). Conversely, the cells with monocyte-like and DC morphology were found to express CD11c (Figure 2l and inset) but not CD163 (Figure 2m). These immunohistochemical findings support the identification of Mϕ and DCs [20] and suggest that the monocyte-like cells are related to DCs.

### Further investigation and enumeration of CD45^+ ^subsets in synovium-rich tissue after adoptive transfer of arthritogenic thoracic duct cells

Importantly, only CD45^+ ^cells were found to express high levels of MHC class II molecules. Thus, by gating MHC II^hi ^cells, it was possible to study the expression of other molecules by this subset, using only two fluorochromes. When aliquots of cells were dual-labelled to detect MHC class II molecules plus either CD11c or CD163, 90% of the MHC II^hi ^cells were found to express CD11c (Figure 2g), 15% expressed CD163 (Figure 2h), and very few cells expressed neither marker. This contrasted with SRT from normal rats (Figure 2c,d), in which the proportions of MHC II^hi ^cells that expressed either CD11c or CD163 were 27% and 30%, respectively, whereas 43% were negative for both markers. Therefore, compared with healthy SRT, there are very few 'indeterminate' cells (expressing neither CD11c nor CD163) in the inflamed SRT.

The maturity of the MHC II^hi ^cells in SRT prepared from normal rats and from rats with adoptively transferred AA was assessed by examining expression of CD36 [24], using mAb UA009 [23]. As shown in Figures 2e and 2i, the molecule was expressed by most of the MHC II^hi ^cells. Thus, relative to DCs in pseudo-afferent lymph, where most of the cells are not stained by mAb UA009 (M. Moghaddami, L.G. Cleland, G. Radisic, G. Mayrhofer, unpublished data), the majority of the MHC II^hi ^cells in SRT have an immature phenotype. However, the proportion of CD36^+ ^cells was slightly higher in SRT from healthy rats (87%) than in SRT from animals 14 days after adoptive transfer (78%). This finding suggests that the proportion of mature DCs is greater in SRT from inflamed paws.

**Figure 3:**
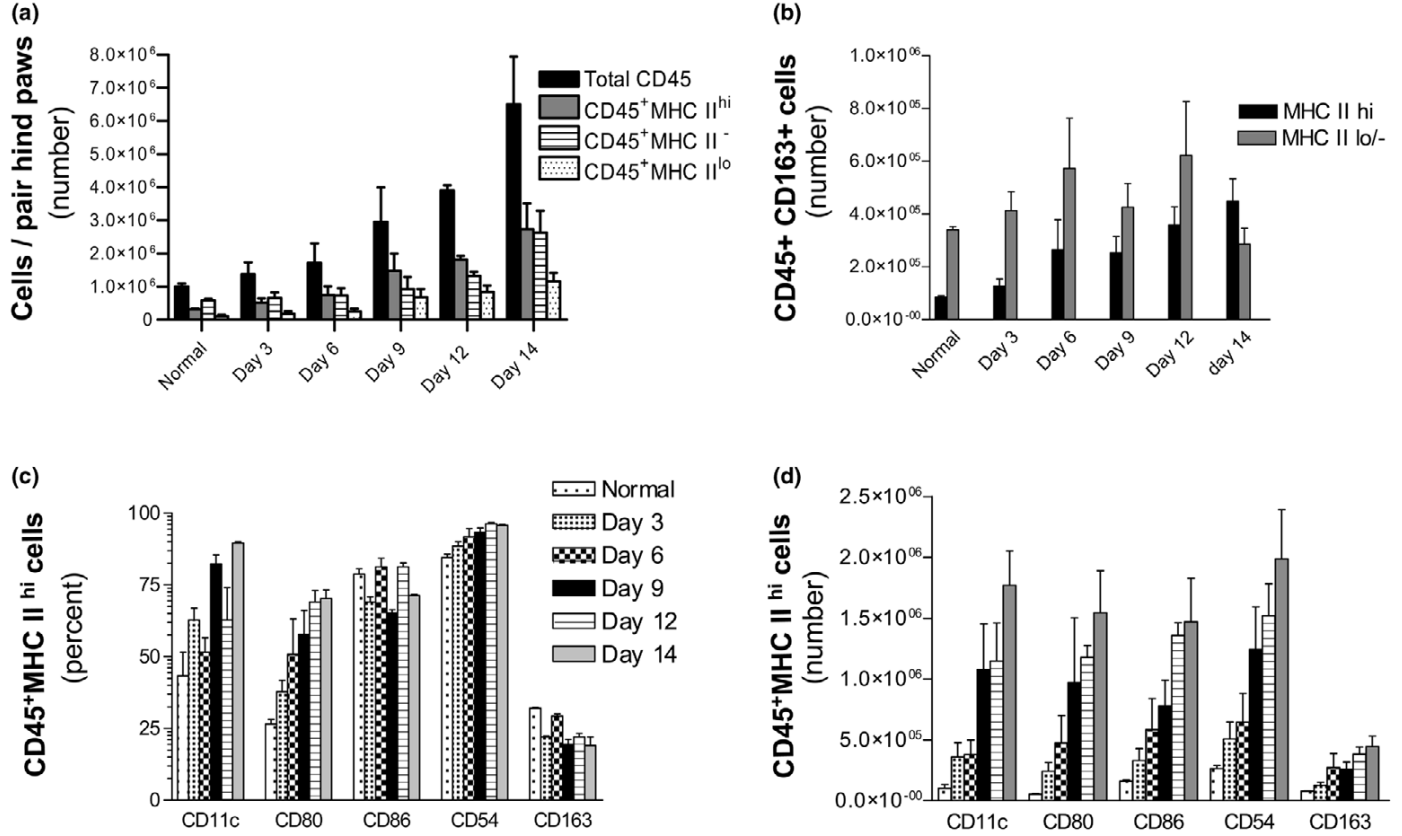
Cell numbers in synovium-rich tissue (SRT) of hind paws during the course of adoptively transferred arthritis. Dual-color flow cytometry was used to analyse cell surface antigens, and CaliBRITE beads were used to estimate cell numbers per pair of hind paws (mean ± standard deviation, *n *= 5). **(a) **Numbers of total CD45^+^, CD45^+^MHC II^hi^, CD45^+^MHC II^lo^, and CD45^+^MHC II^- ^cells in SRT prepared from normal rats and from rats 3 to 14 days after adoptive transfer of arthritis. **(b) **Numbers of MHC II^hi ^and MHC II^lo/- ^macrophages (CD45^+^CD163^+ ^cells) in SRT after adoptive transfer of arthritis. Cell surface antigen phenotype of CD45^+^MHC II^hi ^cells in SRT after adoptive transfer of arthritis, expressed as proportion of cells positive for each marker **(c) **and numbers of cells expressing the markers **(d)**. MHC, major histocompatibility complex.

Incorporation of CaliBRITE beads (Figure 2a) in the analysis [19] allowed the numbers of cells in each subset to be calculated per pair of hind paws (Figure 3a,b,d). The mean total of CD45^+ ^cells in healthy SRT was 1 ± 0.18 × 10^6 ^per pair of hind paws (Figure 3a). In SRT preparations from rats 3, 6, 9, 12, and 14 days after adoptive transfer, the numbers increased slightly during the first 6 days and then rose steeply to reach 6.4 ± 2.8 × 10^6 ^at day 14 after transfer.

MHC II^hi ^cells outnumbered MHC II^lo/int ^cells at all times. The MHC II^hi ^cells had increased approximately 10-fold by day 14 after adoptive transfer (*p *< 0.01 compared with healthy, *p *< 0.05 compared with day 3 or day 6), and MHC II^lo/int ^cells were also more numerous than either in healthy rats (*p *< 0.01) or at days 3 to 6 after transfer (*p *< 0.05) (Figure 3a). Within these subsets, presumptive Mϕ were identified by expression of CD163 (Figure 3b). The numbers of MHC II^hi^CD163^+ ^cells increased progressively over the 14 days after adoptive transfer. Numbers of MHC II^lo/-^CD163^+ ^cells also increased until day 12 but then decreased to levels similar to normal SRT by day 14. Thus, except at day 14, MHC II^lo/- ^Mϕ predominated in both normal and inflamed SRT.

MHC II^- ^cells also increased from approximately day 6 after adoptive transfer and, by day 14, were as numerous as the MHC II^hi ^cells (Figure 3a). As discussed above, most of the MHC II^- ^cells were PMN cells. However, this population also contains presumptive monocytes and the kinetics of recruitment of monocytes and PMN cells are discussed separately below.

In addition, dual-fluorochrome analysis of CD80, CD86, and CD54 expression was performed on the MHC II^hi ^cells (not shown). The proportions of MHC II^hi ^cells that express these markers are shown in Figure 3c. The proportions of CD86^+ ^cells remained relatively constant (70% to 80%) after adoptive transfer, whereas CD54^+ ^cells increased slightly from 84% in healthy SRT to 96% in SRT 14 days after transfer. In contrast, there were significant increases in the proportions of CD11c^+ ^and CD80^+ ^cells over the same period. Importantly, the proportion of CD163^+ ^Mϕ remained unchanged, indicating that the increased proportion of MHC II^+^CD80^+ ^cells must be due to an increase in DC-like cells. This conclusion is supported by a parallel increase in the proportion of CD11c^+ ^cells (Figure 3c). Estimates were also made of the numbers of MHC II^hi ^cells expressing CD11c, CD54, CD80, CD86, and CD163 (Figure 3d). The subsets expressing each of these markers increased during the period following adoptive transfer and for each, the increase was roughly in proportion to the increase in total MHC II^hi ^cells. Whereas there was an increase in Mϕ during adoptively transferred arthritis (Figure 3d), there was no appreciable change in the ratio of MHC II^hi^CD163^+ ^cells (Mϕ) to MHC II^hi^CD163^- ^cells (presumptive DCs) in SRT (Figure 3c).

### Four-fluorochrome analysis of CD11b^+ ^and CD11b^- ^subsets in synovium-rich tissue

A number of subsets of mDCs have been described in rats [25-28]. A CD4^+ ^subset is believed to be stimulatory, whereas it is suggested that a CD4^- ^subset has tolerizing functions [25]. Furthermore, cells that resemble pDCs (MHC II^+^, CD4^+^, CD11b^-^, and CD11c^-^) have been identified [29]. CD11b, which is expressed by the myeloid lineage, distinguishes mDCs from pDCs. Care is needed in making this distinction because, under some circumstances, mDCs may express only low tointermediate levels of CD11b [30,31].

**Figure 4:**
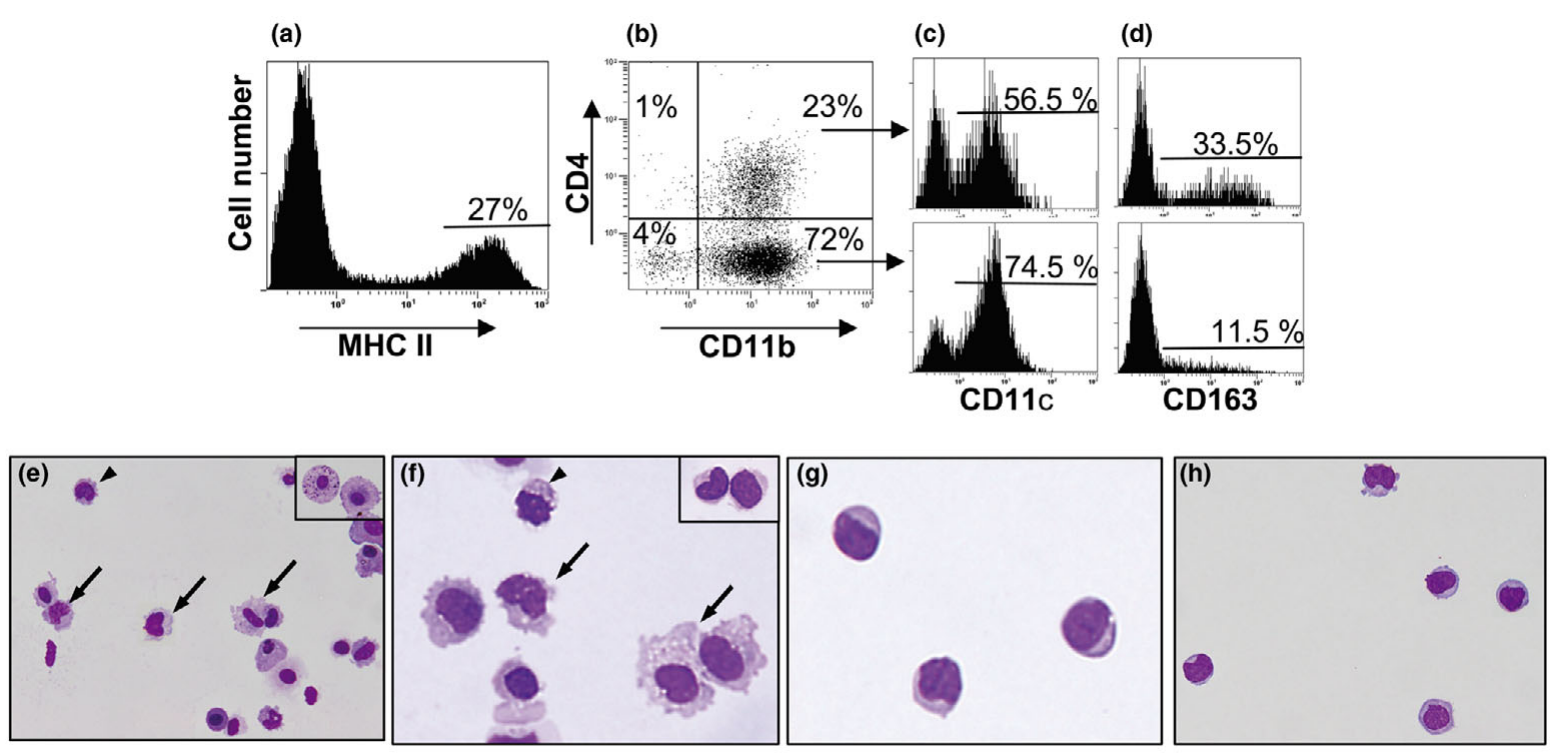
Four-color flow cytometry and cytospin preparations of sorted subsets from synovium-rich tissue 14 days after adoptive transfer. The MHC II^hi ^cells **(a) **were analysed for expression of CD4 and/or CD11b **(b)**. The fourth fluorochrome was used to detect expression of either CD11c **(c) **or CD163 **(d) **by the CD11b^+ ^subsets. **(e) **Giemsa-stained cytospin preparations of the sorted CD11b^+^CD4^+ ^subpopulation consisted mainly of cells with dendritic cell (DC) (arrows) or monocyte-like (arrowhead) morphology with a few cells of typical macrophage morphology (inset). **(f) **Giemsa-stained cytospin preparations of the sorted CD11b^+^CD4^- ^subpopulation consisted mainly of cells with DC (arrows) or monocyte-like (arrowhead and inset) morphology. The sorted CD11b^-^CD4^+ ^**(g) **and CD11b^-^CD4^- ^**(h) **cells had morphology consistent with that described for plasmacytoid DCs. Objective, × 60. MHC, major histocompatibility complex.

Four-color analysis was applied to some of the SRT samples described above (*n *= 2 for each time point) in order to extend the phenotype of the MHC II^hi ^cells isolated from arthritic hind paws. MHC II^hi ^cells (selected as in Figure 4a) could be divided into four subpopulations: CD4^+^CD11b^-^, CD4^-^CD11b^-^, CD4^+^CD11b^+^, and CD4^-^CD11b^+ ^(Figure 4b). Analysis of the CD4^+^CD11b^+ ^and CD4^-^CD11b^+ ^subpopulations showed that both consist mainly of CD11c^+ ^cells (Figure 4c). In the CD4^+^CD11b^+ ^subset, the proportions of CD11c^- ^and CD163^+ ^cells were approximately 43.5% and 33.5%, respectively (Figure 4d), suggesting that most CD11c^- ^cells are CD163^+^MHC II^hi ^Mϕ. This was confirmed using cytospin preparations of sorted CD4^+^CD11b^+ ^cells, in which most had DC-like or monocyte-like morphology (Figure 4e) but some were typical Mϕ (inset). Approximately 10% of CD4^+^CD11b^+ ^cells appear to express neither CD11c nor CD163. Of the CD4^-^CD11b^+ ^cells, most had either DC-like (Figure 4f) or monocyte-like (inset) morphology and most expressed CD11c (75%) and only a few (11.5%) expressed CD163 (Figure 4c,d). As in the case of the CD4^+^CD11b^+ ^subset, a small proportion of MHC II^hi^CD4^-^CD11b^+ ^cells (approximately 14%) did not express either CD11c or CD163 and both subsets contain, therefore, a small number of 'indeterminate' cells.

The subpopulations of MHC II^hi ^cells in the CD11b^- ^quadrants (Figure 4b) do not appear to be of myeloid origin because they do not express even low levels of CD11b. When sorted by fluorescence-activated cell sorting, the morphologies of the CD4^+^CD11b^- ^(approximately 1% of the MHC II^hi ^cells in the DC gate) and CD4^-^CD11b^- ^(approximately 4% of the MHC II^hi ^cells in the DC gate) subsets were similar. Both consist of small- to medium-sized mononuclear cells (Figure 4g,h) resembling pDCs isolated from rat spleen [29]. However, only approximately 5% to 10% express the B220 isoform of CD45 (not shown), which has been described on most rat spleen pDCs [29]. Nevertheless, some of the cells in each subset expressed CD45RC (20% and 40%, respectively), another isoform of CD45 described by the same workers on rat spleen pDCs. Furthermore, approximately half of the cells in these subsets expressed CD86 and some expressed CD80 (20% and 32%, respectively), consistent with at least some being DCs. The CD4^+^CD11b^- ^and CD4^-^CD11b^- ^subsets have similarities in expression of CD11c (75% and 65%), CD54 (70% and 90%), CD172a (60% and 66%), CD11a (60% and 96%), CD32 (50% and 95%), and/or CD5 (20% and 32%). The two subsets also have similarities with putative MHC II^+^CD4^-^CD11b^-^CD11c^+ ^precursors of CD4^+ ^pDCs in mice [32]. Further work is required to determine the functional characteristics of these cells, in particular their response to stimulation with Toll-like receptor 9 (TLR9) agonists [29].

### Changes in numbers of myeloid-derived and CD11b^- ^cells during adoptively transferred arthritis

**Figure 5:**
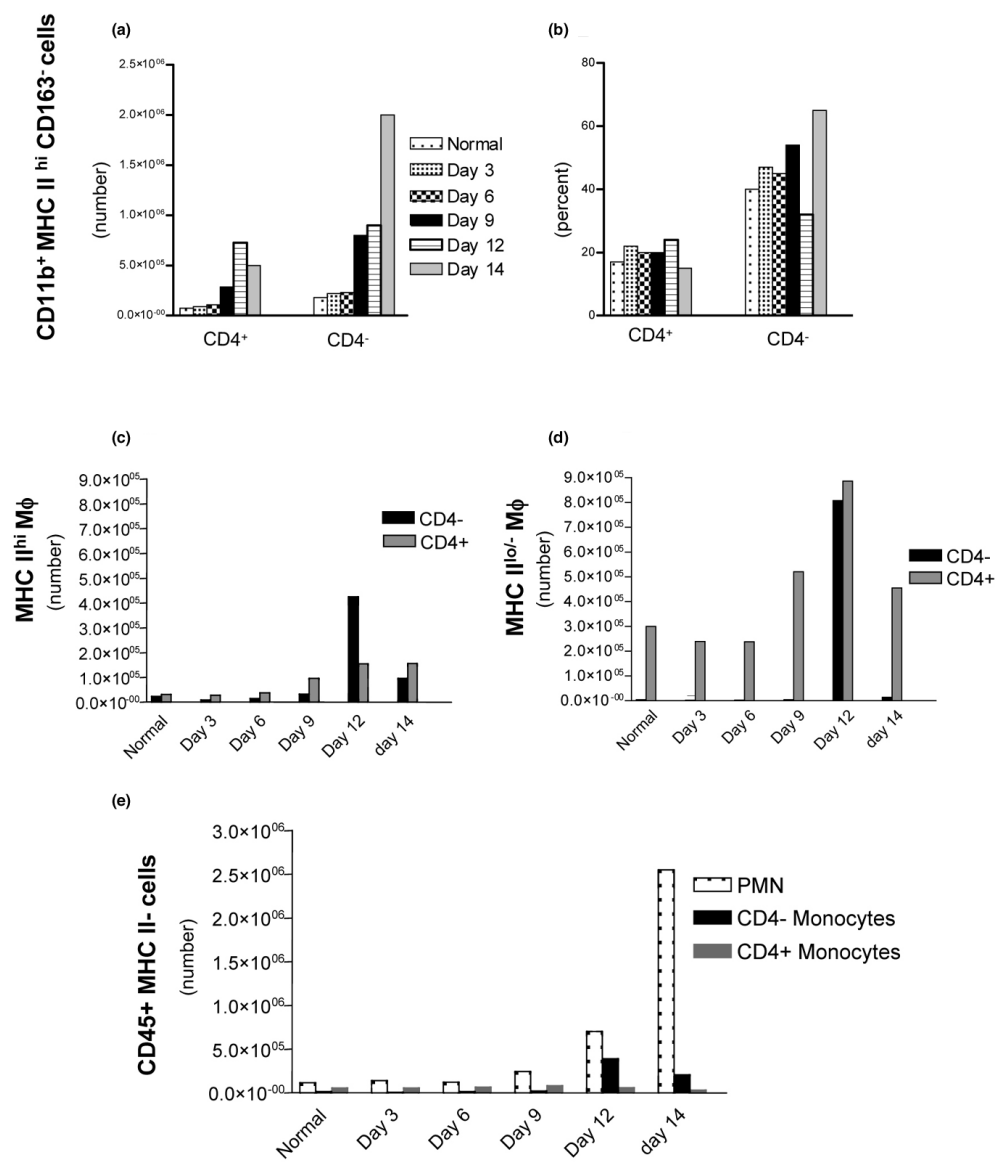
Four-color flow cytometry of subsets from synovium-rich tissue (SRT) following adoptive transfer of arthritis. Cell numbers per pair of hind paws (mean, *n *= 2) were estimated by use of CaliBRITE beads. **(a) **Numbers of putative dendritic cells (MHC II^hi^CD163^- ^cells) with CD4^+^CD11b^+ ^or CD4^-^CD11b^+ ^phenotype. **(b) **Proportions of the same subpopulations. Numbers of CD4^- ^and CD4^+ ^macrophages in the MHC II^hi ^**(c) **and MHC II^lo/- ^**(d) **subpopulations of CD45^+^CD163^+ ^cells. **(e) **Numbers of MHC II^-^CD45^+ ^cells in SRT after adoptive transfer of arthritis. MHC II^- ^cells with the phenotype CD11b^+^CD4^-^CD163^- ^were defined as polymorphonuclear (PMN) cells, whereas those with the phenotypes CD11b^+^CD4^-^CD163^lo ^and CD11b^+^CD4^+^CD163^lo ^were designated as monocytes. MHC, major histocompatibility complex.

As a proportion of CD45^+^MHC II^hi ^cells, the CD4^+^CD11b^+^CD163^- ^and CD4^-^CD11b^+^CD163^- ^subsets remained relatively constant throughout adoptively transferred disease (Figure 5b). However, the numbers of cells in both subsets increased approximately 10-fold by day 14 after transfer (Figure 5a). The numbers of MHC II^hi^CD11b^- ^cells were also estimated (not shown). The CD4^+^CD11b^- ^and CD4^-^CD11b^- ^subsets increased approximately 5- and 10-fold, respectively, by day 14 after transfer but remained unchanged as a proportion of MHC II^hi ^cells (1% and 5%, respectively).

As noted using dual-fluorochrome analysis (Figure 3b), numbers of CD163^+ ^Mϕ increased during adoptively transferred AA. Four-fluorochrome analysis resolved Mϕ into CD4^+ ^and CD4^- ^subsets (Figure 5c,d). In both the MHC II^hi ^and MHC II^lo/- ^subsets, there was a consistent trend toward increased numbers of CD4^- ^Mϕ late in the disease (days 9 to 14), as shown in Figure 5c. The significance of the sharp increase in CD4^+ ^Mϕ at day 12, in both the MHC II^hi ^and MHC II^lo/- ^subsets, is unclear and requires further investigation in additional animals.

To compare the influx of DCs and Mϕ with other inflammatory cells, data from four-fluorochrome analysis were used to identify monocytes (MHC II^-^CD163^lo^) [20] and PMN cells (deduced phenotype MHC II^-^CD11b^+^CD4^-^CD163^-^) amongst the MHC II^- ^cells in SRT. Numbers of PMN cells in normal SRT were small and there were no significant changes during the first 6 days after adoptive transfer (Figure 5e). By day 9, there was a small increase in the numbers of PMN cells and this accelerated rapidly over the following 5 days. These results were consistent with the numbers of PMN cells (CD45^+^MHC II^-^) observed by dual-flurochrome analysis (Figure 3a) and with histological examination of synovial tissue at day 9 after adoptive transfer. Monocytes could be divided into MHC II^-^CD11b^+^CD4^+^CD163^lo ^and MHC II^-^CD11b^+^CD4^-^CD163^lo ^subsets. Only small numbers of CD4^+ ^and CD4^- ^monocytes were observed in healthy SRT. The numbers of CD4^+ ^monocytes did not change significantly throughout adoptively transferred disease (Figure 5e). In contrast, CD4^- ^monocytes increased approximately 30-fold between days 9 and 12 compared with healthy rats and remained at 15-fold that of healthy levels at day 14.

### Surface antigen phenotype of the CD4^+^CD11b^+ ^and CD4^-^CD11b^+ ^subsets of putative myeloid dendritic cells

**Figure 6:**
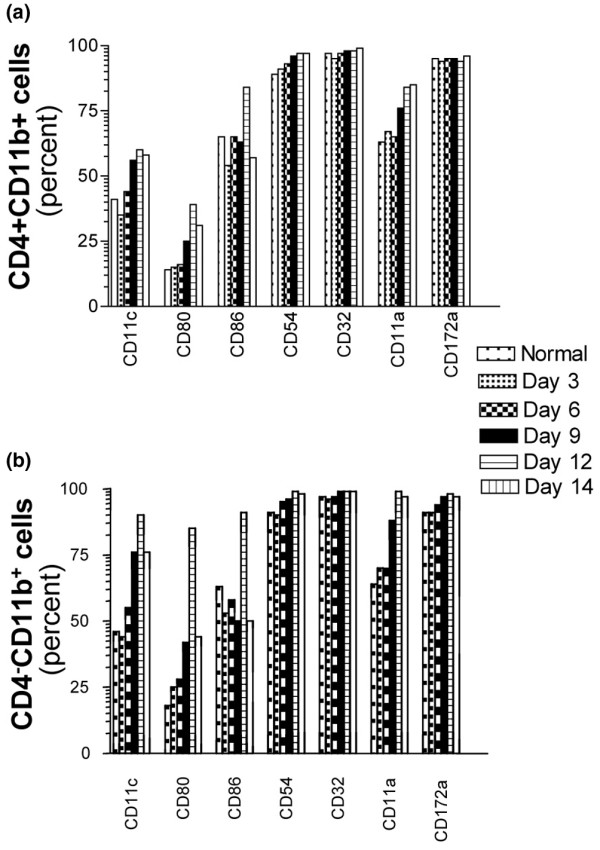
Phenotype of CD4^+^CD11b^+ ^and CD4^-^CD11b^+ ^subpopulations of MHC II^hi ^cells in synovium-rich tissue at indicated times after adoptive transfer of arthritogenic thoracic duct cells. Cell surface phenotype was determined by four-color flow cytometry (Figure 4). The CD4^+ ^**(a) **and CD4^- ^**(b) **subpopulations of MHC II^hi ^CD11b^+ ^cells identified in Figure 4 were assessed by use of a fourth fluorochrome. Proportions of the MHC II^hi ^cells expressing respective surface markers are shown. Means, *n *= 2. MHC, major histocompatibility complex.

Individual markers expressed by the CD4^+^CD11b^+ ^and CD4^-^CD11b^+ ^subsets during the course of adoptively transferred disease are shown in Figure 6. The proportion of cells expressing CD11c increased markedly between days 9 to 14 of the disease (Figure 6a,b). This increase was more pronounced in the CD4^-^CD11b^+ ^subset, in which between 75% and 90% of CD4^-^CD11b^+ ^cells expressed CD11c during this period (Figure 6b) compared with 55% to 60% of the CD4^+^CD11b^+ ^subset (Figure 6a). The proportions of cells expressing CD80 and CD11a also increased during this time in both populations, but the proportion expressing CD86 remained essentially unchanged. At all times, essentially all cells in both subpopulations expressed CD54 and CD32 (Figure 6a,b). In contrast to DCs in afferent lymph [25], the majority of both CD4^- ^and CD4^+ ^cells in SRT expressed CD172a (Figure 6). Interestingly, most of the cells in both subsets expressed CD90 (data not shown).

### Location of putative dendritic cells and macrophages in synovium-rich tissue

**Figure 7:**
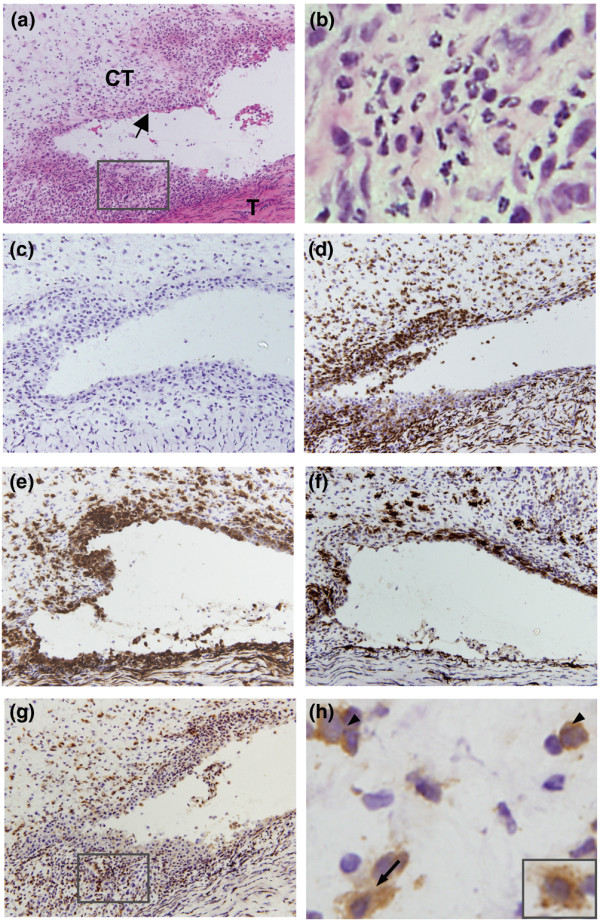
Location and phenotype of putative dendritic cells (DCs) and macrophages in inflamed tissue. Frozen sections prepared from a block of inflamed tissue containing synovium are shown. Tissue was obtained from the lateral side of the ankle 9 days after adoptive transfer of arthritis. **(a) **Frozen section showing inflamed synovial lining (arrow), subintimal connective tissue (CT), and tendon (T), stained with hematoxylin and eosin. **(b) **High-power view of area from box in **(a) **shows polymorphonuclear cells. **(c) **Indirect immunoperoxidase, isotype-matched control monoclonal antibody (mAb). **(d) **Section stained with mAb OX1 showing dense infiltration of the synovial lining and subintimal connective tissue with CD45^+ ^cells. **(e) **Staining with mAb OX6 shows large number of MHC II^+ ^cells in the synovial lining and subintimal connective tissue. **(f) **CD163^+ ^cells stained by mAb ED2 are located mainly in the synovial lining as type A synoviocytes and are present in the subintimal connective tissue in small numbers. **(g) **CD11c^+ ^cells, stained by mAb 8A2, are located mainly in the subintimal connective tissue. **(h) **High-power view of area from box in **(g) **shows CD11c^+ ^monocytes (arrowheads) and DC-like cells (arrow and inset). Objectives, × 60. MHC, major histocompatibility complex.

The soft tissues of the skinned hind paws contain multiple diarthrodial joints and tendon sheaths, with their associated synovial linings and subintimal connective tissues (Figure 7a). However, they also contain tendons, loose areolar connective tissue, adipose tissue, and muscle, plus vascular and nerve tissues. Nine days after adoptive transfer, the subintimal tissues contained many mononuclear cells (Figure 7a) and granulocytes (Figure 7b). Immunohistochemical examination revealed a dense infiltration of the inflamed synovium and surrounding connective tissues with CD45^+ ^cells (Figure 7d), consisting of both mononuclear and PMN leukocytes. Densely stained MHC II^+ ^mononuclear cells (Figure 7e) were present in both the synovial lining and the subintimal tissues, and a large number of lightly stained CD11c^+ ^cells (Figure 7g) were scattered in the subintima. The latter cells exhibited both monocyte and DC morphology (Figure 7h and inset). In contrast, CD163^+ ^cells were fewer in number (Figure 7f) and most were located in the synovial lining (type A synoviocytes), with only scattered cells in the subintimal connective tissue. These observations provide qualitative support to the quantitative studies on inflammatory cells obtained from SRT by enzymatic digestion (Figure 3c). Importantly, they show that Mϕ are a numerically small subset of the total CD45^+ ^cells in the inflamed synovial tissues during adoptively transferred AA whereas MHC II^+ ^and CD11c^+ ^cells are much more abundant.

## Discussion

Adoptive transfer of AA in DA strain rats provides an excellent model in which to study APCs during the effector phase of T cell-mediated autoimmunity. Firstly, activated CD4^+ ^T cells from central lymph of arthritic donors transfer the disease without *ex vivo *stimulation [18] and the cells are recruited selectively to the SRT, where they proliferate [19]. Transfer of AA does not require donor APCs [18], suggesting that APCs in normal synovium are effective in presenting cognate autoantigen(s). Secondly, the enzymatic digestion technique yields a representative population of viable cells from SRT [20,21], allowing examination of cell subsets during adoptively transferred disease and estimation of numbers of cells in the various subsets by calibrated flow cytometry. Estimation of numbers of acute inflammatory cells in SRT allows changes in mononuclear cell subsets to be correlated with other manifestations of inflammation. Importantly, immunohistochemical studies indicate that disaggregated SRT cells are representative of cells within the synovium.

The hind paws were mildly inflamed within 3 to 6 days of transfer of arthritogenic cells, a time when donor T cells are already present in the SRT [19]. Inflammation and total cellularity of SRT increased, reaching sustained levels by days 9 to 12 after transfer. Whereas the numbers of CD45^+ ^cells increased during the first 6 days, PMN cells and typical inflammatory monocytes did not increase until days 9 to 12 and days 12 to 14, respectively. The delayed appearance of MHC II^- ^cells (mainly PMN cells), which comprised approximately 92% of CD45^+^MHC II^- ^cells in SRT by day 14 (Figure 5e), suggests that their recruitment is related indirectly to T cells, possibly involving factors produced by immature and mature DCs [33]. Monocytes, defined herein as circulating non-lymphoid mononuclear cells with the phenotype MHC II^-^CD11c^+^CD163^lo ^[20], did not increase in number until late in the inflammatory process. This apparent delay in recruitment of monocytes may indicate that monocytes that migrate earlier in the disease process express MHC II molecules soon after recruitment, giving rise to the MHC II^hi ^monocyte-like population. The presence of MHC II^hi ^cells with monocyte-like morphology (Figure 2k) which express CD11c (Figure 2l) and CD163 (Figure 2m) supports this notion. Similar MHC II^hi ^monocyte-like cells in SRT from healthy rats are highly endocytic [21], suggesting that this population is functionally similar to DCs. The late rise in MHC II^-^CD163^lo ^monocytes in SRT from rats with adoptively transferred arthritis, therefore, could reflect a decline in factors responsible for upregulating expression of MHC class II molecules. Most of the monocytes in inflamed SRT belong to the CD4^- ^subset, consistent with the description of CD43^lo^CD4^- ^'inflammatory' monocytes in rats [34].

In healthy SRT, approximately 70% of the CD45^+ ^cells had the phenotype MHC II^lo/- ^and most of these were CD163^+ ^Mϕ (Figure 2b–d). Following adoptive transfer, total Mϕ increased approximately two-fold. The proportion of total Mϕ that expressed high levels of MHC class II molecules increased progressively from 20% in normal SRT to 60% at day 14 after adoptive transfer, with the greatest change being in the MHC II^hi ^CD4^- ^subset (Figure 5c). Numbers of MHC II^lo/- ^Mϕ also increased, suggesting that recruitment of new MHC II^lo/- ^Mϕ balances conversion into MHC II^hi ^cells (Figure 3b). Interestingly, the proportions of MHC II^hi^CD163^- ^and MHC II^hi^CD163^+ ^cells remained similar throughout the period of observation (Figure 3c), indicating that control of the commitment to differentiation into DCs or Mϕ was unchanged by the presence of autoreactive T cells.

Total MHC II^hi ^cells in SRT increased 10-fold following transfer of arthritogenic TD cells (Figure 3a). While at least 40% of the MHC II^hi ^cells in healthy SRT are 'indeterminate', expressing neither CD11c nor CD163 [20], the numbers and proportions of CD11c^+ ^cells increased following adoptive transfer (Figure 3c). By day 14, only 10% of the MHC II^hi ^cells did not express CD11c and only 15% expressed CD163 (Figure 2g,h). Therefore, the proportion of 'indeterminate' cells must be less than 10% and the additional MHC II^hi ^cells appear committed to the DC lineage. Increasing numbers of MHC II^hi ^cells expressed the activation markers CD80, CD86, and CD54 (Figure 3d), and the increase in proportion of cells expressing CD80 paralleled the expression of CD11c (Figure 3c). However, the majority of these DC-like cells expressed CD36, indicating that most are not mature DCs [24]. Unlike DCs in mesenteric pseudo-afferent lymph, where most express CD103 (αE2 integrin chain) [35], the vast majority (99%) of MHC II^+ ^cells in SRT lacked this marker (data not shown). Rat DCs are heterogeneous in expression of CD103 [36], and our results indicate that the DC-like cells in SRT resemble veiled cells in rat peripheral afferent lymph, where the majority do not express the molecule (M. Moghaddami, L.G. Cleland, G. Radisic, G. Mayrhofer, unpublished data).

The additional MHC II^hi ^putative DCs in SRT during adoptively transferred disease could reflect increased recruitment of circulating DC precursors [5,37] and/or greater retention of DCs in the tissues [10,11]. Our observations herein, and studies using genetically marked donor monocytes (B. Herdman, M. Moghaddami, G. Mayrhofer, unpublished data), indicate that CD4^- ^monocytes are recruited to the SRT of the hind paws during adoptively transferred AA. Monocytes are activated by exposure to interferon-gamma and during the process of extravasation [38,39]. The activated cells upregulate expression of MHC II molecules and may downregulate CD11c and CD163 [40]. Our results suggest that differentiation of 'indeterminate' cells into DCs is accelerated in tissues undergoing T cell-mediated inflammation. An origin of DCs from monocyte-like cells is consistent with the morphological diversity of the MHC II^hi ^cells from SRT.

The majority of MHC II^hi ^cells in SRT during adoptively transferred disease expressed the myeloid marker CD11b, and of these, most expressed CD11c but not CD163. Only approximately one third of the CD11b^+ ^cells in SRT expressed CD4, showing that the dichotomy of CD4^+ ^and CD4^- ^veiled cells in rat pseudo-afferent lymph [25] exists already in SRT. Whereas numbers of CD4^+ ^and CD4^- ^cells increased after the initiation of disease, the proportions remained relatively constant. Both subpopulations exhibited a progressive increase in the proportion of cells expressing CD80, CD86, and CD11a, and importantly, both expressed CD172a, a molecule expressed only by the CD4^+ ^subset of veiled cells [25]. Moreover, similar proportions of the CD4^+ ^and CD4^- ^subsets in SRT expressed CD90 (data not shown), whereas it is reported that few CD4^- ^CD172a^- ^DCs in rat spleen express CD90 compared with the CD4^+ ^CD172a^+ ^subset [28]. The presence of CD4^- ^CD172a^+^CD90^+ ^cells in SRT from both healthy and arthritic rats indicates that this phenotype is not a response to inflammation. Downregulation of CD172a, therefore, may be an event that precedes migration of CD4^- ^cells from the tissues. Because CD172a has possible co-stimulatory importance [41], its downregulation could signal a switch in the CD4^- ^subset from a stimulatory function to one of maintaining peripheral tolerance [25].

Finally, we have identified MHC II^hi^CD11b^- ^cells in SRT. The CD4^- ^and CD4^+ ^subsets represent only 5% and 1%, respectively, of the total MHC II^hi ^cells in SRT, and although their numbers increased, the relative proportions were similar in healthy and arthritic rats. These cells do not appear to be CD11^lo ^TNF/iNOS-producing DCs of the sort described by Serbina and colleagues [30]. They express high levels of MHC class II molecules, many express the co-stimulatory molecules CD80 and CD86, some express markers such as CD5 and CD45RC associated with rat pDCs [29], and they have a phenotypic resemblance to CD11c^+ ^mouse pDCs [32]. Whereas most do not express the CD45R (B220) isoform of CD45, they do express the CD45RC isoform, which has also been associated with pDCs [29]. Further studies are required to probe the relationship of these cells to pDCs [29], both phenotypically (expression of CD200) and functionally (production of interferon-alpha in response to TLR9 ligands). It is noteworthy that small numbers of pDCs have been described in human rheumatoid synovium [42].

## Conclusion

T cell-induced inflammation in synovium is accompanied by increases in mDCs, Mϕ, and an incompletely characterised subset of MHC II^hi^CD11b^- ^non-lymphoid cells. Further studies are required to determine whether these increases are due to greater precursor recruitment and/or retention and local maturation. The presence of many MHC II^hi ^monocyte-like cells in inflamed SRT suggests that differentiation of monocytes is deviated toward DCs, while the smaller proportion of 'indeterminate' cells and the greater numbers of cells that express co-stimulatory molecules suggest that these cells will be functionally different from those in normal SRT. The large numbers of activated DCs in target tissues of autoimmunity [12,43], therefore, may result from the encounter between autoreactive effector T cells and immature DCs and these cells may exacerbate disease by permitting recursive cycles of T-cell activation.

## Abbreviations

AA = adjuvant-induced arthritis; APC = antigen-presenting cell; CFA = complete Freund's adjuvant; DA = Dark Agouti; DC = dendritic cell; FITC = fluorescein isothiocyanate; Ig = immunoglobulin; Mϕ = macrophage(s); mAb = monoclonal antibody; mDC = myeloid dendritic cell; MHC = major histocompatibility complex; NMS = normal mouse serum; pDC = plasmacytoid dendritic cell; PE = phycoerythrin; PMN = polymorphonuclear; SRT = synovium-rich tissue; TD = thoracic duct; TLR9 = Toll-like receptor 9.

## Competing interests

The authors declare that they have no competing interests.

## Authors' contributions

MM prepared synovium-rich tissue; labelled cells; performed flow cytometry, immunohitochemical analyses, and data analyses; contributed to the design of the experiments; and prepared the first drafts of the manuscript. LC participated in the design of experiments and contributed to finalizing the manuscript. GR performed thoracic duct cannulation and cell injections. GM designed experiments, controlled all experimental steps, performed data analysis, and finalized the manuscript. All authors read and approved the final manuscript.
